# Germline predisposition and somatic mutational landscape in synchronous mucinous metaplasia and neoplasia of the female genital tract

**DOI:** 10.1002/ctm2.70768

**Published:** 2026-08-17

**Authors:** Ying Yuan, Li Zhang, Jiongbo Liao, Bo Wang, Linghui Lu, Dongchen Chu, Yue Shi, Jiaqi Liu, Feng Zhang, Shuyan Tang, Chao Wang

**Affiliations:** ^1^ Obstetrics and Gynecology Hospital, State Key Laboratory of Genetics and Development of Complex Phenotypes, Human Phenome Institute Fudan University Shanghai China; ^2^ School of Clinical Medicine, Shanghai Medical College Fudan University Shanghai China; ^3^ International Peace Maternity and Child Health Hospital, Shanghai Key Laboratory of Embryo Original Diseases Shanghai Jiao Tong University School of Medicine Shanghai China

**Keywords:** *BRCA1*, germline, SMMN‐FGT, somatic, *TP53*

## Abstract

**Background:**

Synchronous mucinous metaplasia and neoplasia of the female genital tract (SMMN‐FGT) with gastric‐type differentiation is a rare disorder with genetic predisposition. Pathogenic *STK11* variants explain only a minority of reported cases, suggesting additional susceptibility genes. However, the genetic landscape of SMMN‐FGT remains poorly characterised.

**Methods:**

Whole‐exome sequencing was performed on 19 lesions and matched normal tissues from 13 cases with SMMN‐FGT. Pathogenic germline variants in cancer‐predisposition genes were identified, and their carrier frequencies were compared with those in common gynaecologic cancers. The somatic mutational landscape of SMMN‐FGT was characterised and compared with conventional cervical cancers. Clonal analyses were performed to identify candidate driver genes, and the functional effects of *KDM5C* were evaluated in vitro using siRNA‐mediated knockdown and plasmid‐mediated overexpression assays.

**Results:**

Germline analysis showed a markedly higher prevalence of pathogenic variants in cancer‐associated genes in SMMN‐FGT (38.5%) than common gynaecologic cancers (4.4%–8.6%). Two cases carried pathogenic *BRCA1* variants, raising the possibility that SMMN‐FGT may be part of a broader *BRCA1*‐associated phenotypic spectrum. Somatic profiling identified *TP53* mutations in five (26.3%) of 19 total lesions, four (80.0%) of which were malignant. Gastric‐type cervical adenocarcinoma (GCA) was the most common malignant subtype of SMMN‐FGT, with *TP53* mutations detected in three (42.9%) of seven GCA lesions and no *PIK3CA* mutations. By contrast, conventional cervical cancers frequently harbour *PIK3CA* mutations (27%–41%), whereas *TP53* mutations are relatively uncommon. Clonal analysis identified *KDM5C* as a candidate driver. *KDM5C* overexpression reduced cell viability, migration and invasion and increased apoptosis, whereas its knockdown produced the opposite effects.

**Conclusions:**

SMMN‐FGT has a distinct genetic profile characterised by an enrichment of pathogenic germline variants, and recurrent *TP53* mutations predominantly affecting malignant lesions. *KDM5C* may function as a candidate tumour suppressor. These findings advance our understanding of SMMN‐FGT pathogenesis and warrant further investigation of its diagnostic and therapeutic implications.

**Key Points**

- SMMN‐FGT exhibits a distinct landscape of germline cancer‐predisposition variants.
- *TP53* mutations are associated with malignant progression in SMMN‐FGT.
- *KDM5C* is identified as a potential tumour suppressor in SMMN‐FGT.

## Graphical abstract

Synchronous mucinous metaplasia and neoplasia of the female genital tract (SMMN‐FGT) is characterised by an enrichment of pathogenic germline variants in cancer‐predisposition genes, particularly *BRCA1* and other homologous recombination repair genes. Recurrent *TP53* mutations are concentrated in malignant lesions, while clonal and functional analyses nominate *KDM5C* as a candidate tumour suppressor. These findings delineate key molecular features of SMMN‐FGT and suggest avenues for future diagnostic and therapeutic research.

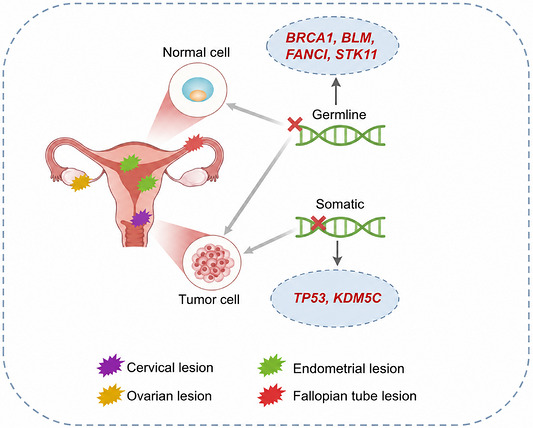

## 1 INTRODUCTION

Synchronous mucinous metaplasia and neoplasia of the female genital tract (SMMN‐FGT) is characterised by simultaneous occurrence of multifocal mucinous lesions involving two or more sites, most commonly the cervix, endometrium, ovaries, and fallopian tubes. SMMN‐FGT is exceedingly rare and was first described by Mikami et al. in 2009.^1^ The lesions span a morphological continuum from benign to malignant forms, including simple gastric metaplasia (SGM), lobular endocervical glandular hyperplasia (LEGH), and gastric‐type mucinous carcinoma (GAS), all of which exhibit gastric‐type differentiation.^2^ Diagnosis is challenging because of the rarity of SMMN‐FGT and its lack of distinctive clinical features, and no consensus has been established on optimal management.^3^

The occurrence of synchronous mucinous lesions across multiple gynaecologic organs suggests a shared etiological basis, potentially involving genetic predisposition. Several cases of SMMN‐FGT have been reported in cases with Peutz–Jeghers syndrome (PJS),^2^ a rare autosomal dominant disorder caused by pathogenic germline variants in the tumour suppressor gene *STK11*.^4^, ^5^ Germline *STK11* variants were identified in cases with both PJS and SMMN‐FGT, supporting a potential pathogenic link.^2^, ^6^ More broadly, pathogenic germline variants in cancer‐predisposition genes are associated with an increased lifetime risk of gynaecologic malignancies.^7^, ^8^ However, genetic testing in SMMN‐FGT has been restricted to *STK11* in cases with clinically diagnosed PJS, leaving the contribution of other germline variants largely unexplored.

Given its extreme rarity, current genetic evidence on SMMN‐FGT is derived largely from isolated case reports.^2^, ^3^, ^9^ Its molecular basis therefore remains poorly defined. Histologically, SMMN‐FGT spans a continuum from metaplastic changes to invasive mucinous adenocarcinoma (MAC), with invasive GAS most commonly arising in the cervix as gastric‐type cervical adenocarcinoma (GCA). GCA is a distinct, HPV‐independent subtype of adenocarcinoma that accounts for nearly two‐thirds of HPV‐independent cervical cancers.^10^ Compared with conventional cervical cancers, GCA exhibits distinct molecular signatures.^11^ Genetic studies have identified *TP53* as the most frequently mutated gene in GCA, followed by *KRAS*, *ERBB2*, *STK11* and *CDKN2A*.^12^, ^13^, ^14^, ^15^, ^16^ By contrast, the molecular profile of gastric‐type lesions outside the cervix remains poorly characterised. Limited case reports have linked somatic mutations in *TP53*, *RB1*, *SMARCB1* and *MLH1* to gastric‐type endometrial adenocarcinoma (GEA).^17^, ^18^ Comprehensive somatic profiling of lesions across multiple sites is therefore needed to clarify the pathogenesis of SMMN‐FGT and to improve its diagnosis.

Whole‐exome sequencing (WES) has become a powerful tool for guiding diagnosis and treatment in clinical practice.^19^ Given the diagnostic complexity of SMMN‐FGT, systematic analysis of germline pathogenic variants in cancer‐susceptibility gene is essential for identifying individuals at increased risk. In parallel, somatic profiling may reveal diagnostic molecular markers and provide potential therapeutic targets. In this study, we applied WES on 19 gastric‐type glandular lesions and matched normal tissues from 13 cases with SMMN‐FGT. By integrating germline and somatic analyses, we aimed to delineate the genomic landscape and molecular pathogenesis of SMMN‐FGT, and provide a basis for more precise diagnosis and clinical management.

## 2 METHODS

### 2.1 Human subjects

Thirteen cases with SMMN‐FGT were enrolled at the Obstetrics and Gynecology Hospital of Fudan University. Each case presented with at least two lesions involving the cervix, endometrium, fallopian tubes or ovaries, all showing gastric‐type differentiation. Two board‐certified pathologists independently confirmed the diagnoses and performed macro‐dissection of tumour regions under microscopic guidance. Based on established clinicopathological criteria, all lesions were pathologically interpreted as independent primary tumours rather than metastatic deposits. Fresh‐frozen or formalin‐fixed paraffin‐embedded (FFPE) lesional tissue and matched normal tissue were collected for DNA extraction.

Haematoxylin and eosin (H&E)‐stained sections were reviewed independently and blindly by two pathologists to confirm a tumour cell content of at least ≥20% in all tumour specimens. Relevant clinical information was retrospectively retrieved from the hospital's electronic medical record system.

### 2.2 DNA extraction and WES

Genomic DNA was extracted from 10‐µm FFPE tissue sections using the AllPrep DNA/RNA FFPE Kit (Qiagen) according to the manufacturer's protocol. DNA concentration and purity were assessed by NanoDrop 2000 spectrophotometry (Thermo Fisher Scientific) based on A260/A280 ratios. DNA integrity was evaluated by electrophoresis on 1% agarose gels.

Sequencing libraries were constructed using the KAPA HyperPrep Kit (Roche Sequencing Solutions). Exome capture was performed using the SureSelect Human All Exon V6 system (Agilent Technologies), followed by 150 bp paired‐end sequencing on the Illumina NextSeq 500 platform (Illumina Inc.). Raw sequencing reads were processed with Fastp (v0.23.2)^20^ for quality control. High‐quality reads were aligned to GRCh37 (GCF_000001405.13) reference genome using BWA‐MEM (v0.7.17).^21^ PCR duplicates were removed with Picard MarkDuplicates (http://broadinstitute.github.io/picard/). Local realignment around insertions and deletions (indels) and base quality recalibration were performed using Genome Analysis Toolkit (gatk;v4.6.0.0).^22^

### 2.3 Identification of germline variants

Germline variants were called using GATK *HaplotypeCaller*. High‐confidence variants were retained if they had a population allele frequency ≤1%, sequencing depth ≥5×, and variant allele frequency ≥25%. Variants were first called in Genomic Variant Call Format (GVCF) mode, followed by recalibration with Variant Recalibrator and Apply VQSR to improve accuracy for single‐nucleotide variants and small indels. Functional annotation was performed using the Variant Effect Predictor (VEP),^23^ and ClinVar annotations were used to prioritise variants classified as pathogenic or likely pathogenic.

Downstream analysis was focused on 73 genes associated with gynaecologic cancers (Table S1). These genes represent a clinically curated subset derived from the union of three established clinical resources: the ACMG recommendations for clinically actionable secondary findings (28 genes), the National Comprehensive Cancer Network (NCCN) guidelines for hereditary cancer risk assessment (33 genes), and the BROCA Cancer Risk Panel (University of Washington), a clinically validated germline testing platform for hereditary cancer predisposition (60 genes). Any gene included in at least one of these three resources was incorporated into our analysis, yielding a final panel of 73 unique genes. We subjected non‐synonymous coding variants to additional quality control and minor allele frequency (MAF) assessment. Variants were classified according to ACMG guidelines into five categories: pathogenic, likely pathogenic, variant of uncertain significance, likely benign and benign. Classification incorporated evidence from ClinVar, Genome Aggregation Database (gnomAD), and the Human Gene Mutation Database (HGMD), with ClinVar serving as the primary reference for missense variants.

### 2.4 Identification of somatic mutations

Somatic mutations were identified using MuTect2 (v3.4‐0),^22^ MuSE,^24^ and VarScan2 (v2.4.2).^25^ To ensure high‐confidence calls, we applied the following filters: sequencing depth >10×; variant allele frequency >3% in tumour samples; corresponding allele frequency <1% in matched normal samples (when available). Variants present in the 1000 Genomes Project or Exome Aggregation Consortium databases were excluded. Only mutations detected by at least two of the three variant callers (MuTect2, MuSE, VarScan2). The resulting variants were functionally annotated using Ensembl VEP and converted to MAF format with vcf2maf for downstream analysis.

### 2.5 Processing of UK Biobank data

UK Biobank WES data were analysed to compare genetic pathogenic variants with gynaecologic tumours. The analysis included only female participants diagnosed with gynaecological cancers, defined by ICD‐10 codes C53, C54, C56 and C57. We obtained variant call format (VCF) files containing WES data for these samples. Variant filtering and annotation followed the same criteria as in the study cohort germline analysis.

### 2.6 Processing of TCGA data

To compare the mutational profile of SMMN‐FGT with conventional cervical cancers, we retrieved somatic MAF files and associated clinical information from the TCGA‐CESC (TCGA‐CESC) cohort. Based on the primary diagnosis field, cases were stratified into squamous cell carcinoma, adenocarcinoma and serous carcinoma subgroups. We performed comparative mutational profiling using the R package maftools (v2.18.0).^26^

### 2.7 Mutational burden and signature analyses

Mutational analyses were performed in R (v4.4.1) using Bioconductor packages. Tumour mutational burden (TMB) was calculated using maftools (v2.18.0) and defined as the number of non‐synonymous somatic mutations per megabase of callable coding sequence captured by the WES assay. Mutational signatures were analysed using MutationalPatterns (v3.14.0). Somatic single‐nucleotide variants were assigned to 96 trinucleotide substitution contexts, and their contributions were estimated by refitting the data to the COSMIC single‐base substitution signatures (v3.3). We specifically quantified the contributions of the homologous recombination deficiency‐associated signature SBS3 and the APOBEC‐associated signatures SBS2 and SBS13.

### 2.8 Subclone analysis

Subclonal architecture was analysed using PyClone, a Bayesian modelling framework that clusters mutations based on their CP, integrating allele frequencies and copy number variations. Single‐nucleotide variants, copy number alterations, and sample‐specific parameters were jointly analysed to infer clonal hierarchies and the evolutionary relationships among subclones. This analysis was performed to explore the potential clonal relatedness and evolutionary trajectories of anatomically distinct lesions within individual cases, as well as to identify shared and lesion‐specific driver mutations associated with tumour evolution.^27^, ^28^

### 2.9 Cell culture and transfection

C33A cells were obtained from the American Type Culture Collection and cultured in Dulbecco's modified Eagle medium (DMEM) supplemented with 10% foetal bovine serum (FBS) and 1% penicillin–streptomycin–amphotericin B. Cells were maintained at 37°C in a humidified atmosphere containing 5% CO_2_. At 70%–80% confluence, cells were transfected using Lipofectamine 3000 (Thermo Fisher Scientific). For *KDM5C* knockdown, cells were transfected with 50 nM control siRNA (siCTRL) or one of two *KDM5C*‐targeting siRNAs (siKDM5C#1 and siKDM5C#2). For *KDM5C* overexpression, cells were transfected with the pCMV‐3′FLAG‐KDM5C plasmid or the corresponding empty vector using Lipofectamine 3000 and P3000 reagents according to the manufacturer's instructions. The siRNAs and plasmids were purchased from YiXueSheng Biosciences. Cells were harvested or reseeded for downstream assays 24 h after transfection. Knockdown and overexpression efficiency was confirmed by reverse transcription quantitative PCR (RT‐qPCR).

### 2.10 Quantitative real‐time PCR

Total RNA was isolated from C33A cells and reverse‐transcribed into cDNA using PrimeScript RT Master Mix (Takara, RR036A). Quantitative real‐time PCR was performed using TB Green Premix Ex Taq (Takara, RR420A). β‐Actin was used as the internal control. Relative *KDM5C* mRNA expression was calculated using the 2−ΔΔCt method. Primer sequences are listed in Table S2.

### 2.11 Cell proliferation assay

Cell proliferation was evaluated using the Cell Counting Kit‐8 assay (CCK‐8; Vazyme). Briefly, 24 h after transfection, 3500 C33A cells were seeded into 96‐well plates per well. At the indicated time points, 10 µL CCK‐8 reagent was added to each well, followed by incubation at 37°C for 2–4 h. The absorbance at 450 nm was measured using a BioTek microplate reader.

### 2.12 Wound‐healing assay

Cell migration was assessed using a wound‐healing assay. At 24 h after transfection, C33A cells were seeded into six‐well plates at a density of approximately 1 × 10^6^ cells per well and cultured until a confluent monolayer was formed. A linear scratch was generated using a sterile pipette tip, and detached cells were removed by washing with phosphate‐buffered saline (PBS). Cells were then cultured in serum‐free DMEM. Images were captured at 0 and 24 h using an Olympus microscope at 4× magnification. Wound closure was quantified using ImageJ.

### 2.13 Transwell invasion assay

Cell invasion was examined using 24‐well Transwell chambers with 8‐µm pore membranes. The upper chambers were coated with NEST Matrigel diluted at a ratio of 1:75 in serum‐free DMEM and incubated at 37°C for 1 h. The chambers were then hydrated with serum‐free DMEM before cell seeding. At 24 h after transfection, C33A cells were resuspended in serum‐free DMEM and seeded into the upper chambers at a density of 2 × 10^5^ cells per chamber. The lower chambers were filled with 800 µL DMEM containing 12.5% FBS. After incubation for 24 h, invaded cells on the lower surface were fixed with 4% paraformaldehyde for 15–30 min, stained with  .1% crystal violet for 30 min, washed with PBS, and photographed using an Olympus microscope at 10× magnification.

### 2.14 Cell apoptosis assay

Cell apoptosis was assessed using an Annexin V‐FITC/PI apoptosis detection kit (Novonesis) according to the manufacturer's instructions. Cells were harvested 24 h after transfection, washed with PBS, stained with Annexin V‐FITC and PI in binding buffer, and analysed using a CytoFLEX flow cytometer. Flow cytometry data were analysed using CytExpert software and plotted using FlowJo. Flow cytometry data were analysed using CytExpert software and plotted using FlowJo.

### 2.15 Cell‐cycle analysis

Cell‐cycle distribution was analysed using a cell‐cycle detection kit (Novonesis) according to the manufacturer's protocol. At 24 h after transfection, C33A cells were collected and fixed with pre‐chilled 75% ethanol at 4°C for 24 h. Then, cells were washed with PBS and incubated with PI/RNase staining solution at 37°C for 30 min in the dark. DNA content was detected using a CytoFLEX flow cytometer. The percentages of cells in the G1, S and G2/M phases were analysed using CytExpert software and plotted using FlowJo.

### 2.16 Statistics

Differences in pathogenic variant carrier frequencies between independent groups were assessed using two‐sided Fisher's exact test. Comparisons of continuous outcomes from the in vitro experiments were performed using two‐tailed Student's *t*‐test. A *p* value of <.05 was considered statistically significant.

## 3 RESULT

### 3.1 Clinical characteristics of the cases with SMMN‐FGT

**FIGURE 1:**
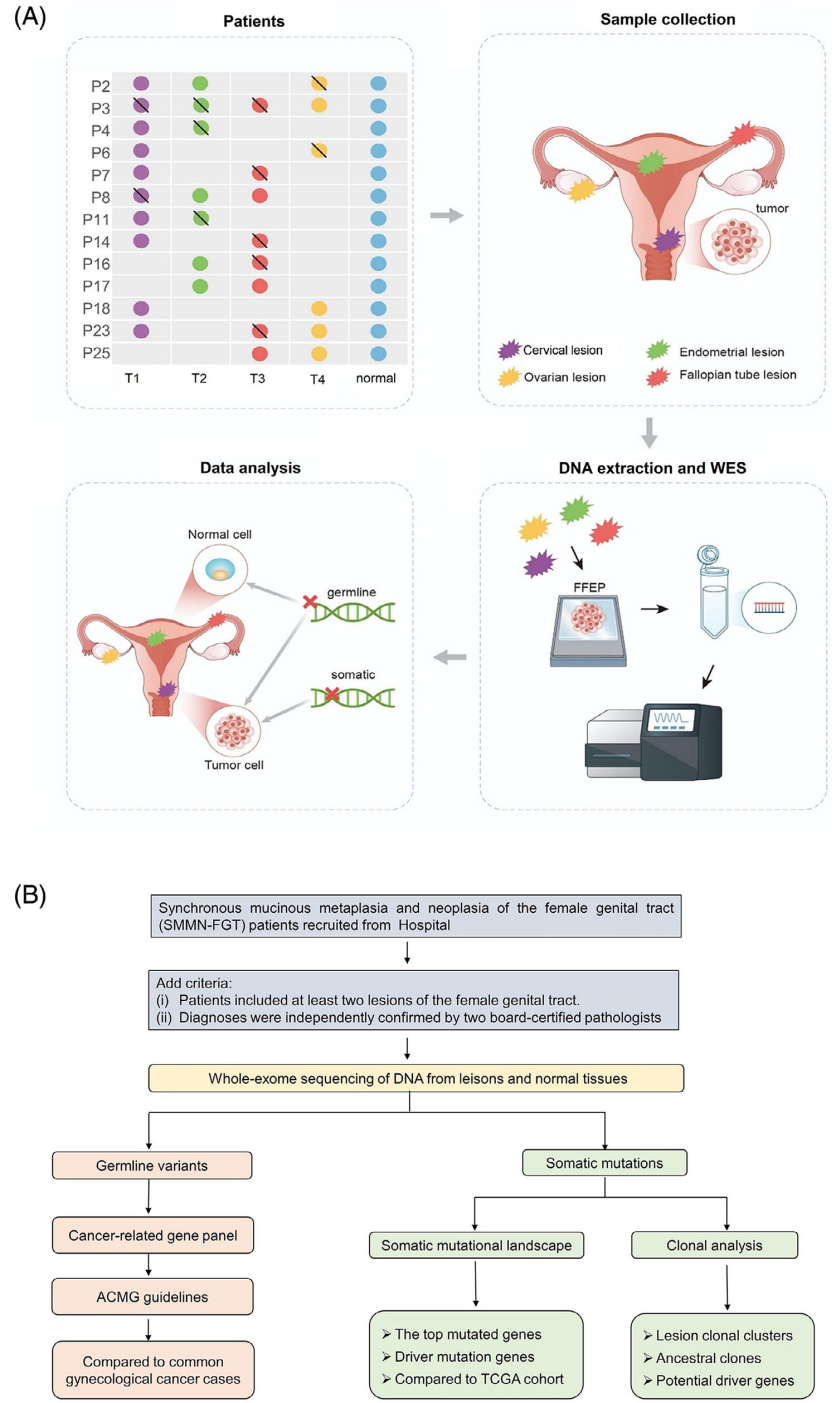
Overview of study samples and analytical workflow. (A) Anatomic sources of the analysed lesions and the experimental workflow. (B) Overall study design and bioinformatic analysis pipeline. T1, cervical lesion; T2, endometrial lesion; T3, fallopian tube lesion; T4, ovarian lesion.

The clinical features of the 13 cases with SMMN‐FGT are detailed in Table S3. The median age at sample collection was 49 years (range, 37–83 years). HPV testing was negative in 84.6% (11/13) of cases, including all seven individuals diagnosed with GCA. The lesions spanned the histologic spectrum from non‐neoplastic and benign to borderline and malignant. Nine cases had invasive GAS, whereas the remaining four cases presented with benign or transitional lesions, including LEGH, SGM, borderline mucinous tumours (BMT) and MAC. A total of 19 lesions were collected and categorised for downstream analyses: eight cervical lesions (T1), four endometrial lesions (T2), three fallopian tube lesions (T3), and four ovarian lesions (T4; Figure 1A). Matched non‐neoplastic control tissues were sampled from histologically normal cervical epithelium, whose distinct anatomic boundaries allowed precise sampling with minimal contamination from adjacent tissue. Among the cohort, six cases contributed lesions from two distinct gynaecologic sites, whereas the remaining seven cases provided a single lesion. The overall analytical pipeline and study design and analytical workflow are shown in Figure 1B.

### 3.2 Pathogenic germline *BRCA1* variants recurrently identified in SMMN‐FGT

After stringent filtering, 22 209 high‐confidence germline variants were identified across the 13 cases, most of which were missense variants (Figure S1A). Of these, 268 variants mapped to 71 cancer‐predisposition genes, including five pathogenic variants and 50 variants of uncertain significance, classified according to American College of Medical Genetics and Genomics (ACMG) guidelines (Figure S1B,C). Overall, five cases (38.46%) carried germline pathogenic variants in cancer‐susceptibility genes (Table 1); four frameshift and one splice‐site variant. Remarkably, two cases (P16 and P17) carried pathogenic *BRCA1* variants (NM_007300.4: c.5533_5540del, p.Ile1845Aspfs; NM_007300.4: c.1360_1361del, p.Ser454Terfs). Both cases developed GAS involving the endometrium and fallopian tube, despite having no personal history of breast or ovarian cancer. The family history of P16 included lung cancer with her father and ovarian cancer in her sister. These cases expand the known phenotypic spectrum of *BRCA1*‐associated malignancies, suggesting that *BRCA1* variants may predispose to SMMN‐FGT.

**TABLE 1:** Molecular characteristics of pathogenic germline variants in synchronous mucinous metaplasia and neoplasia of the female genital tract (SMMN‐FGT) cases.

Gene	Case	Genotype	Function	American College of Medical Genetics and Genomics (ACMG) classification	cDNA change	Protein change
*BLM*	P6	Homozygote	Splice site	Pathogenic	NM_001287246.2: c.3558+2T>C	Splice
*FANCI*	P7	Heterozygote	Frameshift deletion	Pathogenic	NM_001376911.1:c.3118_3119del	NP_001363840.1:p.Lys1040GlufsTer8
*STK11*	P8	Heterozygote	Frameshift deletion	Pathogenic	NM_000455.5:c.652del	NP_000446.1:p.Ala218LeufsTer69
*BRCA1*	P16	Heterozygote	Frameshift deletion	Pathogenic	NM_007300.4:c.5533_5540del	NP_009231.2:p.Ile1845AspfsTer3
*BRCA1*	P17	Heterozygote	Frameshift deletion	Pathogenic	NM_007300.4:c.1360_1361del	NP_009231.2:p.Ser454Ter

Pathogenic variants in two additional homologous recombination repair genes, *BLM* and *FANCI*, were identified. Case P6 carried a homozygous *BLM* variant (NM_001287246.2: c.3558+2T>C) and had GCA with a left‐sided ovarian mucinous cystadenoma and a right‐sided BMT. ClinVar associates this variant with Bloom syndrome (accession RCV001219429.8), an autosomal recessive disorder characterised by growth restriction, diabetes, and broad cancer predisposition (OMIM 210900). Consistent with this, P6 exhibited short stature (1.52 m) and a longstanding history of diabetes. Case P7 carried a *FANCI* variant (NM_001376911.1: c.3118_3119del) and developed had GCA with SGM in the oviduct. *FANCI* variants are associated with Fanconi anaemia, and P7 had a history of hypothyroidism, which may reflect underlying Fanconi syndrome. These findings suggest that inherited defects in homologous recombination repair genes may contribute to SMMN‐FGT susceptibility.

To determine whether pathogenic germline variants are enriched in SMMN‐FGT, we compared their prevalence with that in cases with common gynaecologic cancers in the UK Biobank.^29^ We evaluated germline pathogenic or likely pathogenic (P/LP) variants in cases with cervical, endometrial, fallopian tube, and ovarian cancers, including squamous cell carcinoma, adenocarcinoma, and serous subtypes. In 540 cervical cancer cases from the UK Biobank, 21 pathogenic variants across 17 genes were identified in 4.4% (24/540) of cases (Table S4). In contrast, 38.46% (5/13) of SMMN‐FGT cases carried P/LP germline variants, representing a highly significant enrichment compared with cervical cancer (38.46% vs. 4.4%; *p* = .000225, odds ratio = 14.05, two‐sided Fisher's exact test). Higher carrier frequencies were also observed relative to endometrial cancer (38.46% vs. 6.9%, *p* = .001333, odds ratio = 8.45), ovarian cancer (38.46% vs. 6.5%, *p* = .001106, odds ratio = 8.92), and fallopian tube cancer (38.46% vs. 8.6%, *p* = .0073, odds ratio = 6.56). These results suggest that pathogenic germline variants may be enriched in SMMN‐FGT and support a potential role for germline genetic predisposition in its pathogenesis.

### 3.3 *TP53* dominates the somatic mutational landscape of SMMN‐FGT

**FIGURE 2:**
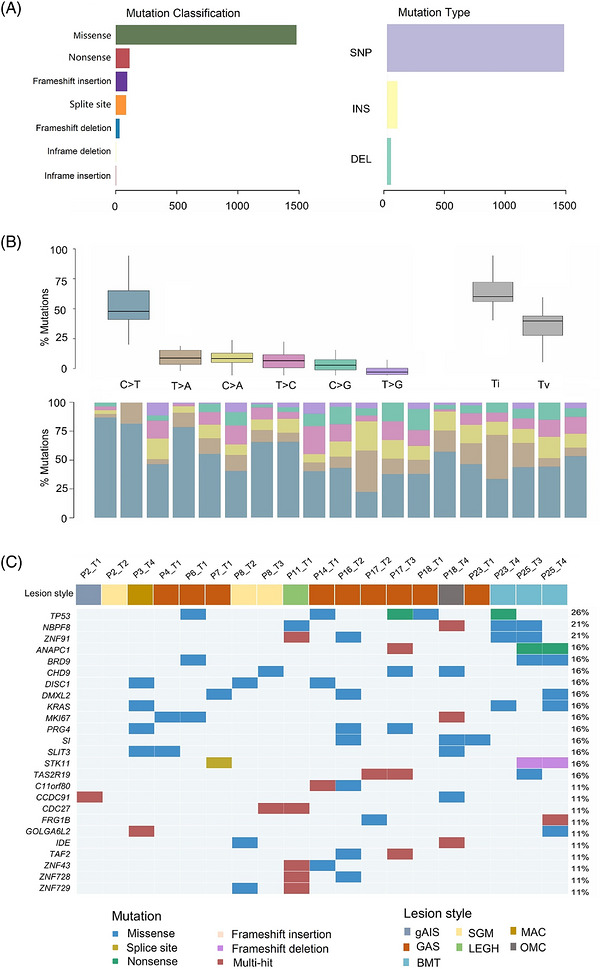
Somatic mutational landscape of synchronous mucinous metaplasia and neoplasia of the female genital tract (SMMN‐FGT) lesions. (A) Distribution of somatic variants according to functional class. (B) Distribution of single‐nucleotide variant substitution classes and the transition‐to‐transversion ratio. (C) Oncoplot of the most frequently mutated genes across 19 lesions from 13 cases. Columns represent lesions, rows represent genes, and colours denote variant classes as indicated in the key. ALEGH, atypical lobular endocervical glandular hyperplasia; BMT, borderline mucinous tumour; gAIS, gastric‐type mucinous carcinoma in situ; GAS, gastric‐type mucinous carcinoma; LEGH, lobular endocervical glandular hyperplasia; MAC, mucinous adenocarcinoma; MDA, minimal deviation adenocarcinoma; OMC, ovarian mucinous cystadenoma; SGM, simple gastric‐type mucinous metaplasia.

Across the 19 SMMN‐FGT lesions, missense mutations were the most common class of somatic alteration (Figure 2A). Among somatic single‐nucleotide variants (SNPs), transitions (Ti) were more frequently than transversions (Tv), with C>T transitions representing the most common type (Figure 2B). Somatic profiling across the 13 cases (Figure 2C) showed that *TP53* was the most frequently mutated gene, occurring in five of 19 lesions (26%), predominantly in malignant samples. Notably, four of these five *TP53*‐mutant lesions were malignant GAS, whereas the remaining lesion was an ovarian BMT. *NBPF8* and *ZNF9*1 were each mutated in 21% of lesions, and *KRAS* mutations were detected in 16%.

Somatic variants were annotated using the Catalogue of Somatic Mutations in Cancer (COSMIC) and ClinVar (Table S5). *TP53* had the largest number of mutations annotated as pathogenic or likely pathogenic, affecting four cases: two lesions were GCA, one was GAS in the oviduct and one was a BMT in the ovary. The predominance of *TP53* mutations in malignant lesions suggests that they may contribute to malignant progression. A pathogenic stop–gain mutation in *CDKN2A* was classified as pathogenic in an ovarian MAC. *STK11* mutations were identified in three of 19 lesions (16%), including two BMTs and one LEGH lesion. These results highlight recurrent somatic alterations that may contribute to tumour initiation and progression in SMMN‐FGT.

**FIGURE 3:**
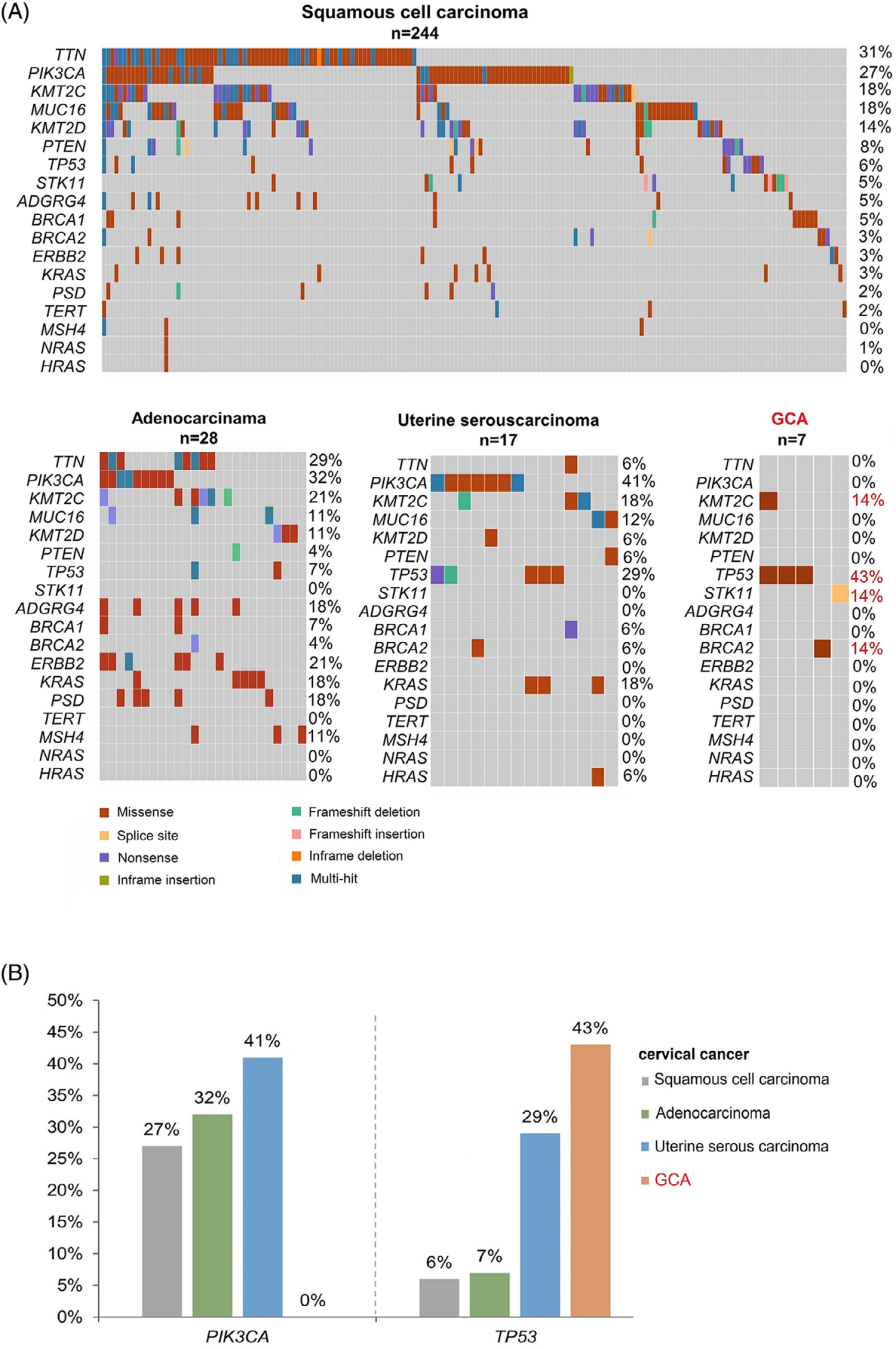
Comparison of somatic alterations across cervical cancer subtypes. (A) Oncoplot comparing somatic alterations in synchronous mucinous metaplasia and neoplasia of the female genital tract (SMMN‐FGT)‐associated GCA with those in cervical cancer subtypes from the TCGA‐CESC cohort. (B) Frequencies of *PIK3CA* and *TP53* mutations across cervical cancer subtypes.

To compare the genomic distinction between GCA within SMMN‐FGT and conventional cervical cancers, we analysed somatic mutation data from 289 cases in the TCGA‐CESC cohort, including squamous cell carcinoma, adenocarcinoma and serous carcinoma subtypes, retrieved from The Cancer Genome Atlas (TCGA). Our analysis revealed marked differences in the distribution of somatic mutations across histologic subtypes (Figure 3A). Well‐established cancer driver genes, including *TP53*, *BRCA2*, *KMT2C* and *STK11*, were mutated across multiple subtypes. Notably, *PIK3CA* mutations were prevalent in squamous cell carcinoma, adenocarcinoma and serous carcinoma but were entirely absent in GCA. In contrast, *TP53* mutations were most frequent in GCA, occurring in 43% of lesions, compared with 29% in serous carcinoma and only 6%–7% in squamous cell carcinoma and adenocarcinoma (Figure 3B).

To assess potential confounding by HPV status, the TCGA‐CESC cohort was stratified by HPV status. Only six HPV‐negative cases were available, and their overall mutational profile was broadly similar to that of the full TCGA‐CESC cohort. *PIK3CA* and *TP53* mutations were detected in 67% and 33% of these HPV‐negative cases, respectively (Figure S2). Although this analysis was limited by the small number of HPV‐negative cases, the results suggest that the differences in *TP53* and *PIK3CA* mutation frequencies between GCA and conventional cervical cancer cannot be attributed solely to HPV status.

### 3.4 Tumour mutational burden and mutational signatures

SMMN‐FGT exhibited an intermediate TMB relative to the TCGA pan‐cancer cohorts (Figure S3A). No statistically significant differences in TMB were detected according to anatomic site or the presence of pathogenic germline variants in homologous recombination repair genes (Figure S3B,C). Mutational signature analysis showed minimal contributions from the APOBEC‐associated signatures SBS2 and SBS13. By contrast, substantial contributions from the homologous recombination deficiency‐associated signature SBS3 were observed in five lesions: P2_T2, P16_T2, P17_T2, P17_T3 and P23_T1 (Figure S4). The lesion from P16 and both lesions from P17 exhibited SBS3, and both cases carried pathogenic germline *BRCA1* variants. This concordance supports an association between germline *BRCA1* variants and homologous recombination deficiency in these lesions. SBS3 was also detected in lesions from P2 and P23, suggesting that additional mechanisms may underlie homologous recombination deficiency in SMMN‐FGT.

### 3.5 *KDM5C* is a potential driver gene in SMMN‐FGT

**FIGURE 4:**
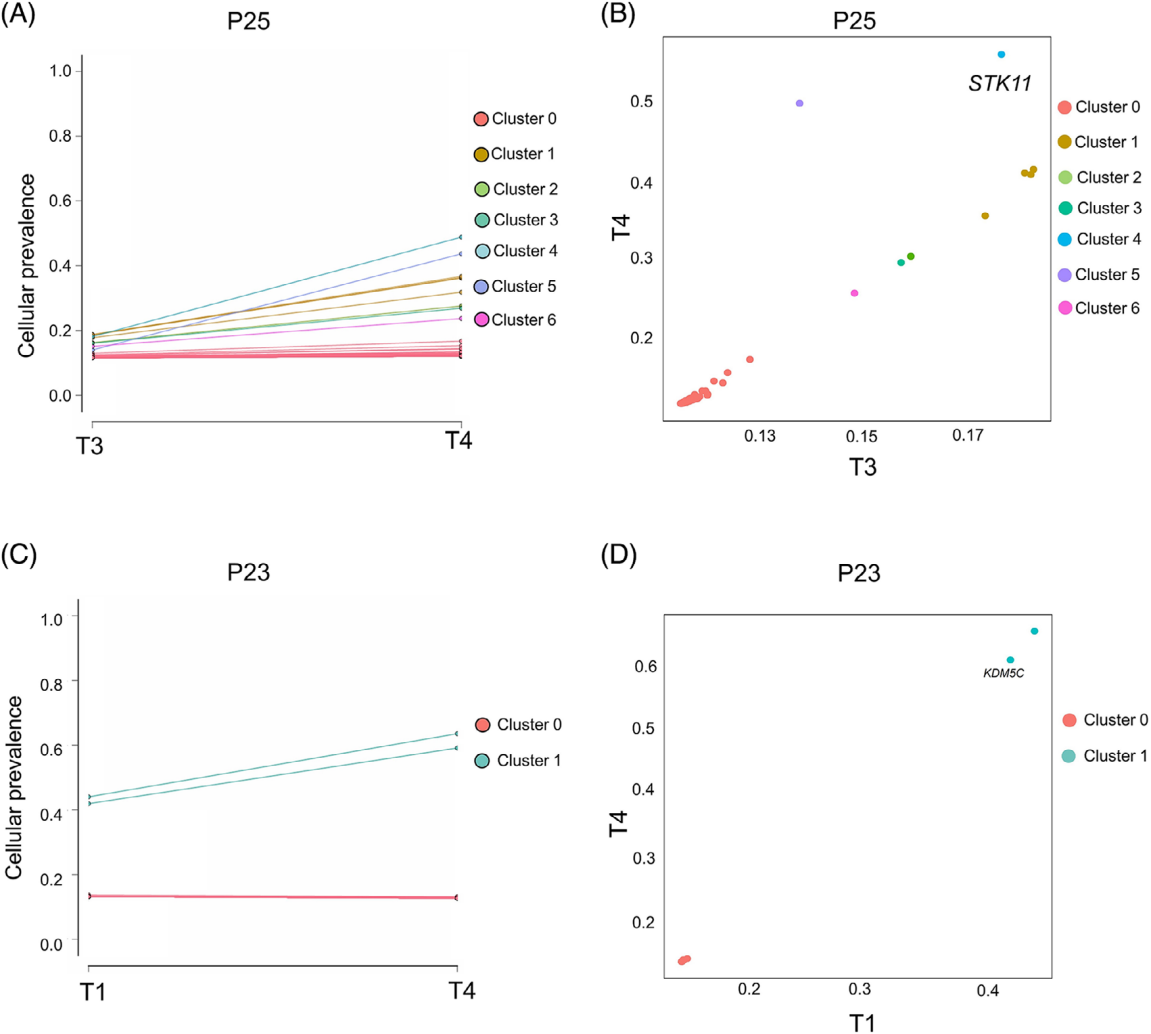
Clonal architecture of lesions from cases P25 and P23. (A, C) Parallel‐coordinate plots showing the cellular prevalence (CP) of PyClone‐defined clusters across paired lesions from P25 (A) and P23 (C). The *x*‐axis indicates lesion site, and the *y*‐axis indicates CP. (B, D) Scatterplots comparing mutation‐level CP between the two distinct lesions from P25 (B) and P23 (D). Each point represents a somatic mutation, and the *x*‐ and *y*‐axes indicate its CP in the two lesions from the same case. Colours are consistent across panels for each case and denote distinct PyClone‐defined clusters. T1, cervical lesion; T3, fallopian tube lesion; T4, ovarian lesion.

Determining whether multiple lesions in the same case share a common driver mutation provides insights into tumour evolution and potential therapy resistance.^30^ PyClone was used to estimate cellular prevalence (CP) for each mutation and assign mutations to distinct clonal clusters.^31^ Six cases had lesions from two different organs, and clonal analysis was performed for five cases, excluding P2 due to insufficient sequencing depth. Among these, P8, P17 and P23 (Figure 4C) each displayed two clonal clusters; P18 had three clusters; and P25 (Figure 4A) had seven clusters. Notably, in P8, P17, and P18, all clusters exhibited low CP values, with no shared high‐CP ancestral clone across lesions. These observations are consistent with the histopathological diagnosis, compatible with the interpretation that these lesions may represent independent primary tumours rather than metastatic dissemination. However, given the limited sampling and the low CP of some subclones, the clonal evolution analysis should be considered exploratory and interpreted cautiously.

Candidate driver mutations were then examined within higher prevalence clusters. In P25, cluster 2 in the ovarian lesion had a CP of  .55 and harboured an *STK11* mutation (Figure 4B). In contrast, all clonal populations in the fallopian tube lesion had CP values below  .2 (Figure 4B), suggesting that the ovarian and fallopian tube lesions may arise from distinct driver events. In case P23, cluster 1 displayed higher CP values than cluster 0 in both the cervical and ovarian lesions, indicating that cluster 1 likely represents the ancestral clone (Figure 4C). This cluster harboured mutations in *KDM5C* and *PRKDC* (Figure 4D). The presence of these mutations in both lesions suggests that they may represent shared early events. Given its established tumour‐suppressive function, *KDM5C* was prioritised for functional evaluation.

### 3.6 *KDM5C* suppresses malignant phenotypes in C33A cells

**FIGURE 5:**
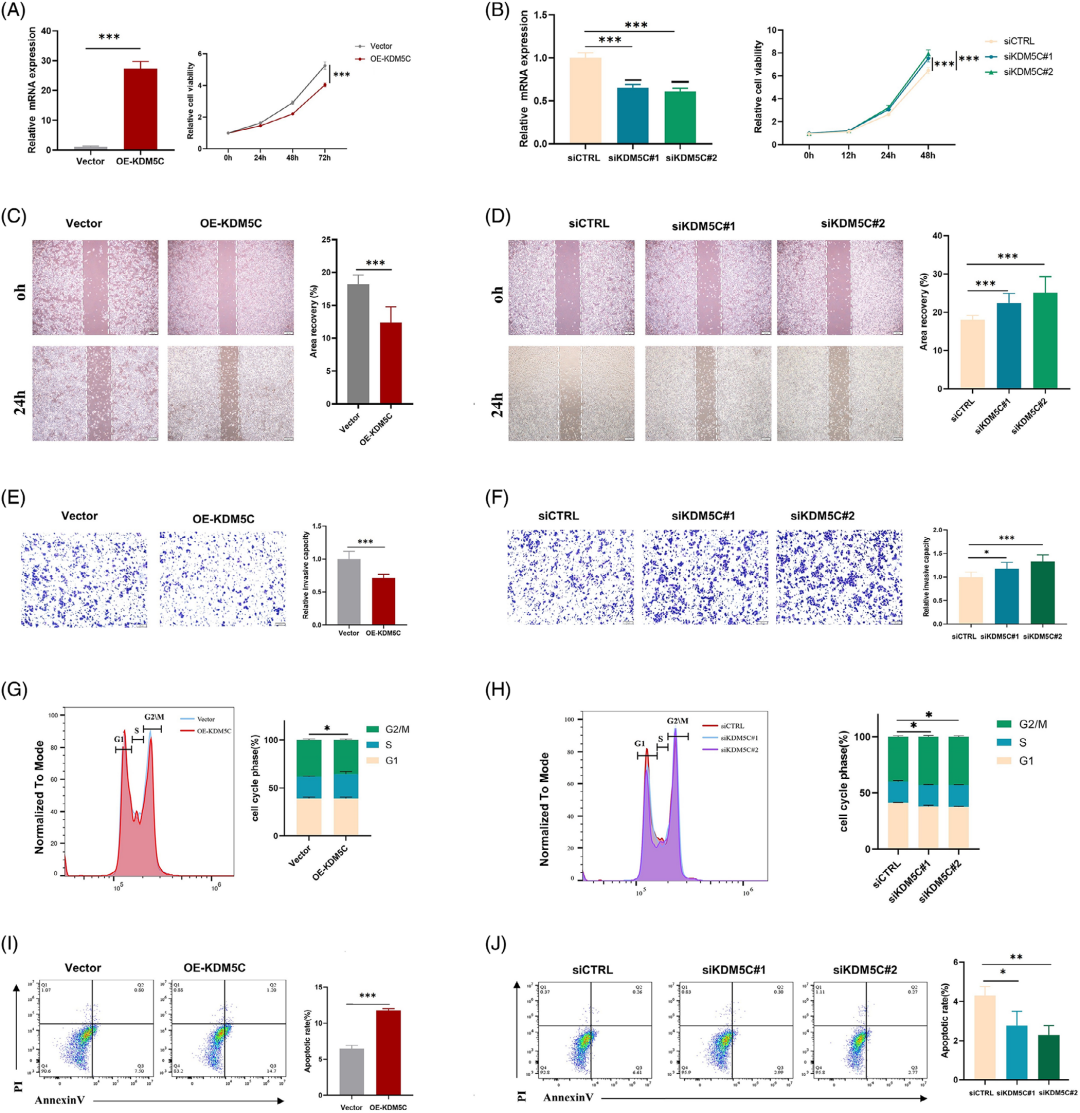
Functional effects of *KDM5C* modulation in C33A cells. (A, B) qRT‐PCR validation of *KDM5C* overexpression and knockdown efficiency, and the corresponding effects on C33A cell proliferation determined by CCK‐8 assays in the (A) OE‐KDM5C and (B) siKDM5C groups compared with their respective controls (*n* = 5). (C, D) Representative images (left) and quantification (right) of wound‐healing assays performed 24 h after scratch generation in C33A cells following (C) *KDM5C* overexpression and (D) knockdown. Cell migration was quantified as the percentage of wound area recovery (*n* = 3). (E, F) Representative images (left) and quantitative analysis (right) of Transwell invasion assays following (E) *KDM5C* overexpression and (F) knockdown (*n* = 3). (G, H) Cell‐cycle distribution analysed by flow cytometry (left) and quantification of cells in the G1, S, and G2/M phases (right) in the (G) OE‐KDM5C and (H) siKDM5C groups (*n* = 3). (I, J) Representative flow cytometry plots of Annexin V‐FITC/PI staining (left) and quantification of total apoptotic cells (right) following (I) *KDM5C* overexpression and (J) knockdown (*n* = 3). *n* refers to biological replicates. Data are presented as mean ± SD. Statistical significance was determined using an unpaired two‐tailed Student's *t*‐test. *p < .05, **p < .01, ***p < .001 versus the corresponding control group.

The functional effects of *KDM5C* were examined in the HPV‐negative cervical cancer cell line C33A, which shares an HPV‐independent molecular background with SMMN‐FGT. The qRT‐PCR assay confirmed successful transfection, with *KDM5C* mRNA expression increasing 27‐fold following overexpression (*p* = 6.76 × 10^−^^6^) and decreasing to 60%–65% of control levels after siRNA‐mediated knockdown (*p* = 5.27 × 10^−^^5^, *p* = 2.95 × 10^−^^5^, respectively; Figure 5A,B).

CCK‐8 assays demonstrated that *KDM5C* overexpression reduced cell viability by 23.2% at 72 h (*p* = 6.65 × 10^−^^6^), whereas *KDM5C* knockdown increased cell viability by 16.5% and 22.7% in the siKDM5C#1 and siKDM5C#2 groups (*p* = 4.49 × 10^−^^4^ and 7.50 × 10^−^^5^; Figure 5A,B). In wound‐healing assays, overexpression reduced wound closure by 25.5% (*p* = 1.13 × 10^−^^5^), whereas knockdown increased wound closure 1.24‐fold with either siRNA (*p* = 1.57 × 10^−^^4^ and 1.97 × 10^−^^4^, respectively; Figure 5C,D). Similarly, Transwell assays revealed that *KDM5C* overexpression reduced invasive capacity by 25% (*p* = 2.89 × 10^−^^4^), whereas knockdown enhanced invasion by 21% and 31% in siKDM5C#1 and siKDM5C#2 cells, respectively (*p* = .0311 and 8.82 × 10^−^^4^; Figure 5E,F).

Flow cytometric analysis further showed that *KDM5C* overexpression induced S‐phase arrest, increasing the S‐phase population by 12.2% while reducing the G2/M fraction by 7.1% (*p* = .0367; Figure 5G). In contrast, *KDM5C* knockdown promoted cell‐cycle progression, increasing the G2/M population by 1.08‐fold in both siKDM5C#1 and siKDM5C#2 cells (*p* = .0313 and  .0160, respectively; Figure 5H). Consistent with these findings, *KDM5C* overexpression increased the total apoptotic rate by 83% (*p* = 6.35 × 10^−^^5^), whereas knockdown reduced apoptosis by 35% and 46% in the siKDM5C#1 and siKDM5C#2 groups, respectively (*p* = .0384 and  .00666; Figure 5I,J). Collectively, these findings demonstrate that *KDM5C* suppresses cervical cancer cell proliferation, migration, and invasion while inducing S‐phase arrest and apoptosis, supporting its tumour‐suppressive role in SMMN‐FGT.

## 4 DISCUSSION

In this study, we analysed 19 gastric‐type mucinous lesions from 13 cases with SMMN‐FGT to characterise their genetic landscape and molecular basis. Previous studies of common gynaecologic cancers, including ovarian, fallopian tube, and cervical tumours, have reported pathogenic germline variants in cancer‐associated genes at frequencies ranging from 4% to 24%.^32^, ^33^, ^34^ Similarly, our analysis of UK Biobank data showed a frequency of 4.4%–8.6% for pathogenic germline variants in these common gynaecologic cancers. In contrast, pathogenic germline variants were identified in five of 13 cases with SMMN‐FGT (38.5%). Although this finding should be interpreted cautiously given the small cohort, it suggests that germline cancer‐predisposition variants may be enriched in SMMN‐FGT and may contribute to susceptibility to multifocal disease.

Women with PJS are predisposed to mucinous lesions throughout the female genital tract, as well as gynaecologic tumours such as ovarian sex cord tumours with annular tubules and mucinous ovarian tumours.^35^, ^36^ *STK11* (also known as *LKB1*) is a highly conserved serine/threonine kinase and a critical tumour suppressor implicated in multiple cancers.^4^, ^37^ In our cohort, one case with clinically diagnosed PJS carried a pathogenic germline *STK11* variant, consistent with the reported association between PJS and SMMN‐FGT. This observation supports increased awareness of SMMN‐FGT during the gynaecologic evaluation of women with PJS and consideration of germline *STK11* testing in cases with SMMN‐FGT, particularly when clinical features of PJS are present.

*BRCA1*, *FANCI* and *BLM* genes are a well‐established mechanism driving genomic instability, which increased predisposition to multiple primary malignancies.^38^, ^39^, ^40^ SMMN‐FGT is characterised by multifocal gastric‐type mucinous carcinoma arising throughout the female genital tract, germline of DNA Damage Repair genes deficiency may promote tumourigenesis through genomic instability across multiple sites of the female genital tract. In our cohort, we identified two cases with SMMN‐FGT carrying pathogenic germline variants in *BRCA1*, both of whom developed GAS in the endometrium and fallopian tubes. Previous genetic studies of SMMN‐FGT have primarily focused on targeted *STK11* testing rather than comprehensive analyses of other cancer‐predisposition genes. Large *BRCA1* carrier cohort suggested that *BRCA1* are strongly associated with increased risks of ovarian, breast, and endometrial cancers,^41^, ^42^, ^43^ but there is currently no direct evidence linking *BRCA1* variants to SMMN‐FGT, likely because of the extreme rarity of this disease. Our findings expand the spectrum of *BRCA1*‐associated malignancies and suggest that *BRCA1* pathogenic variants may also predispose carriers to SMMN‐FGT. Furthermore, *BRCA1* variant carriers may benefit therapeutically from poly‐ADP ribose polymerase (PARP) inhibitors,^44^ offering a targeted treatment strategy for cases with these pathogenic variants. However, given the generally favourable early prognosis of SMMN‐FGT and the current duration of case follow‐up, the practical efficacy of *BRCA1* as a predictive biomarker for targeted therapies warrants long‐term clinical observation.

SMMN‐FGT is a rare entity whose somatic mutational landscape remains poorly characterised. When malignant, its gastric‐type mucinous lesions most often present as GAS, usually in the cervix and less commonly in the endometrium or fallopian tube. Prior genomic studies of GCA have identified recurrent alterations in *TP53*, *CDKN2A*, *KRAS* and *STK11*, with *TP53* mutations detected in 41% of cases.^12^ A separate analysis of 21 primary GCA tumours reported *TP53* alterations in 52.4% of samples.^16^ Data on GAS arising outside the cervix are limited; however, a study of 27 GEA also identified *TP53* as the most frequently mutated gene.^18^ In our cohort, *TP53* mutations were detected in five of 19 lesions (26%); four were malignant GAS, whereas the fifth was an ovarian BMT. This pattern is broadly consistent with previous findings in GCA and GEA and suggests that *TP53* mutations may represent a shared molecular feature of gastric‐type mucinous carcinoma across gynaecologic sites. Their preferential occurrence in malignant lesions further suggests a role in malignant progression, although larger longitudinal studies are required to establish their prognostic or diagnostic value.

GCA of SMMN‐FGT is an HPV‐independent cervical adenocarcinoma characterised by prominent gastric‐type differentiation. Comparison with the TCGA‐CESC cohort showed that PIK3CA mutations were common in conventional cervical cancer subtypes, particularly squamous cell carcinoma (41%), but were not detected in GCA. Conversely, *TP53* was the most frequently mutated gene in GCA. *PIK3CA* is a central component of the PI3K/AKT pathway, which regulates cellular proliferation, survival, and apoptosis^45^ and its activation has been implicated in HPV‐associated cervical tumourigenesis.^46^, ^47^ However, *PIK3CA* mutations were also frequent in the small HPV‐negative TCGA subgroup, indicating that HPV status alone does not explain the difference between GCA and conventional cervical cancer. Collectively, the contrasting *TP53* and *PIK3CA* mutation patterns support a distinct molecular pathogenesis for GCA arising in SMMN‐FGT. Abnormal p53 immunostaining and targeted sequencing of *TP53* and *PIK3CA* may complement morphologic and immunophenotypic assessment, but their diagnostic utility requires validation in larger independent cohorts.

Clonal expansion driven by heterogeneous tumour subpopulations is a well‐recognised driver of intra‐tumoural diversity.^31^ To investigate this process, we analysed clonal architecture in five cases from whom two independent gastric‐type glandular lesions were available. In case P23, *KDM5C* mutations were identified in the ancestral clone shared by both cervical and ovarian lesions. *KDM5C* encodes a histone H3K4 demethylase and is frequently mutated across multiple cancer types.^48^, ^49^ Functionally, *KDM5C* has been implicated in preserving genomic stability through maintenance of heterochromatin integrity, thereby exerting tumour‐suppressive effects. In addition, *KDM5C* represses oncogenic transcriptional programs by reducing H3K4me3 levels at target promoters, leading to silencing of pro‐tumourigenic pathways.^50^, ^51^ Consistent with these tumour‐suppressive functions, *KDM5C* overexpression reduced cell viability, migration and invasion and increased apoptosis in HPV‐negative C33A cells, whereas *KDM5C* knockdown produced the opposite effects. The presence of a *KDM5C* alteration in a shared high‐prevalence cluster raises the possibility that it represents an early event in lesion development. Together, the clonal and functional findings nominate *KDM5C* as a candidate tumour‐suppressor gene in SMMN‐FGT.

In conclusion, we used WES to characterise the germline and somatic genomic landscape of 19 gastric‐type lesions from 13 cases with SMMN‐FGT. Our findings suggest an enrichment of pathogenic germline variants in cancer‐predisposition genes, identify recurrent *TP53* mutations preferentially affecting malignant lesions, and nominate *KDM5C* as a candidate tumour‐suppressor gene. Together, these results provide an initial molecular framework for understanding the pathogenesis of SMMN‐FGT.

Several limitations should be acknowledged. First, the rarity and diagnostic complexity of SMMN‐FGT limited the cohort size and the availability of multiple lesions from individual cases, and no independent validation cohort was available. Larger multicentre studies are therefore needed to confirm these findings. Second, WES has limited sensitivity for structural variants and non‐coding alterations; complementary approaches, including long‐read sequencing, may provide a more complete view of the genomic landscape. Third, the functional experiments relied on an HPV‐negative cervical cancer cell line rather than a disease‐specific model and did not directly evaluate the case‐derived *KDM5C* variant. Developing SMMN‐FGT cell lines, organoids, and other representative models will be important for mechanistic studies. Finally, the retrospective and exploratory nature of this study precludes immediate clinical application. Prospective validation is required to determine the diagnostic value of *TP53* mutations and whether *BRCA1* or other homologous recombination repair alterations confer clinically relevant therapeutic vulnerabilities.

## AUTHOR CONTRIBUTIONS

Chao Wang, Shuyan Tang and Ying Yuan conceptualised the study and developed the methodology. Chao Wang, Jiongbo Liao, Bo Wang and Yue Shi collected biomaterials and clinical data. Li Zhang and Jiaqi Liu performed the experiments. Linghui Lu and Dongchen Chu reviewed all available tumours blocks and pathology reports. Shuyan Tang and Ying Yuan performed curated data and developed the pipeline for WES. Chao Wang, Shuyan Tang and Feng Zhang oversaw the analyses and contributed to data interpretation. Ying Yuan wrote the original draft. Shuyan Tang, Feng Zhang and Chao Wang revised the manuscript. All the authors reviewed, edited and approved the final version of the manuscript.

## CONFLICT OF INTEREST STATEMENT

The authors declare no conflicts of interest.

## ETHICS STATEMENT

All human subject research followed the ethical principles outlined in the Declaration of Helsinki (2013). Written informed consent was obtained from all participants prior to enrolment. The study protocol was reviewed and approved by the Ethics Committee of the Obstetrics and Gynecology Hospital of Fudan University (approval number: 2025‐142).

## Supporting information



Supporting Information

Supporting Information

Supporting Information

Supporting Information

Supporting Information

Supporting Information

Supporting Information

Supporting Information

Supporting Information

## Data Availability

The data presented here will be available upon request to the corresponding author (wang1980-55@163.com). Remaining data are available in the UK Biobank and TCGA databases.
