# Supplementary material for: Germline predisposition and somatic mutational landscape in synchronous mucinous metaplasia and neoplasia of the female genital tract
Source: Clin Transl Med. 2026 Aug 17;16(8):e70768. doi: 10.1002/ctm2.70768 (PMC13478790; doi:10.1002/ctm2.70768)
Supplement: Supplementary file 1 [file CTM2-16-e70768-s001.docx]

Table S2. Primer and siRNA Sequences

| **Name** | **Sequence (5′ to 3′)** |
| --- | --- |
| KDM5C-F | GGGTCCGACGATTTTCCTACC |
| KDM5C-R | GCGATGGGCCCTGATTTTCG |
| ACTB-F | CTGGAACGGTGAAGGTGACA |
| ACTB-R | AAGGGACTTCCTGTAACAACGCA |
| siKDM5C-1 sense | GAGCUGCUGCAGCAACUAATT |
| siKDM5C-1 antisense | UUAGUUGCUGCAGCAGCUCTT |
| siKDM5C-2 sense | GACAGUAAACGGCACCUAATT |
| siKDM5C-2 antisense | UUAGGUGCCGUUUACUGUCTT |
