# Supplementary material for: Germline predisposition and somatic mutational landscape in synchronous mucinous metaplasia and neoplasia of the female genital tract
Source: Clin Transl Med. 2026 Aug 17;16(8):e70768. doi: 10.1002/ctm2.70768 (PMC13478790; doi:10.1002/ctm2.70768)
Supplement: Supplementary file 2 [file CTM2-16-e70768-s002.docx]

| Table S1 The list of 73 genes associated with gynecological cancer | |
| --- | --- |
| Gene | Source |
| *ALK* | BROCA Cancer Risk Panel |
| *APC* | ACMG; BROCA Cancer Risk Panel |
| *ATM* | NCCN; BROCA Cancer Risk Panel |
| *ATR* | BROCA Cancer Risk Panel |
| *AXIN2* | NCCN; BROCA Cancer Risk Panel |
| *BAP1* | NCCN; BROCA Cancer Risk Panel |
| *BARD1* | NCCN; BROCA Cancer Risk Panel |
| *BMPR1A* | ACMG; BROCA Cancer Risk Panel |
| *BRCA1* | ACMG; BROCA Cancer Risk Panel |
| *BRCA2* | ACMG; BROCA Cancer Risk Panel |
| *BRIP1* | NCCN; BROCA Cancer Risk Panel |
| *CDH1* | NCCN; BROCA Cancer Risk Panel |
| *CDK4* | NCCN; BROCA Cancer Risk Panel |
| *CDKN2A* | NCCN; BROCA Cancer Risk Panel |
| *CEBPA* | NCCN |
| *CHEK2* | NCCN; BROCA Cancer Risk Panel |
| *CTNNA1* | BROCA Cancer Risk Panel |
| *DICER1* | NCCN; BROCA Cancer Risk Panel |
| *EGFR* | NCCN |
| *FANCM* | BROCA Cancer Risk Panel |
| *FH* | NCCN; BROCA Cancer Risk Panel |
| *FLCN* | NCCN; BROCA Cancer Risk Panel |
| *GEN1* | BROCA Cancer Risk Panel |
| *GREM1* | NCCN; BROCA Cancer Risk Panel |
| *HOXB13* | BROCA Cancer Risk Panel |
| *KIT* | NCCN |
| *LZTR1* | NCCN |
| *MAX* | ACMG |
| *MEN1* | ACMG; BROCA Cancer Risk Panel |
| *MET* | NCCN; BROCA Cancer Risk Panel |
| *MITF* | NCCN; BROCA Cancer Risk Panel |
| *MLH1* | ACMG; BROCA Cancer Risk Panel |
| *MLH3* | NCCN; BROCA Cancer Risk Panel |
| *MSH2* | ACMG |
| *MSH3* | NCCN; BROCA Cancer Risk Panel |
| *MSH6* | ACMG; BROCA Cancer Risk Panel |
| *MUTYH* | ACMG; BROCA Cancer Risk Panel |
| *NBN* | BROCA Cancer Risk Panel |
| *NF1* | NCCN; BROCA Cancer Risk Panel |
| *NF2* | ACMG; BROCA Cancer Risk Panel |
| Gene | Source |
| *NTHL1* | NCCN; BROCA Cancer Risk Panel |
| *PALB2* | ACMG; BROCA Cancer Risk Panel |
| *PIK3CA* | BROCA Cancer Risk Panel |
| *PMS2* | ACMG; BROCA Cancer Risk Panel |
| *POLD1* | NCCN; BROCA Cancer Risk Panel |
| *POLE* | NCCN; BROCA Cancer Risk Panel |
| *POT1* | NCCN; BROCA Cancer Risk Panel |
| *PRKAR1A* | BROCA Cancer Risk Panel |
| *PTCH1* | NCCN; BROCA Cancer Risk Panel |
| *PTEN* | ACMG; BROCA Cancer Risk Panel |
| *RAD51B* | BROCA Cancer Risk Panel |
| *RAD51C* | NCCN; BROCA Cancer Risk Panel |
| *RAD51D* | NCCN; BROCA Cancer Risk Panel |
| *RB1* | ACMG; BROCA Cancer Risk Panel |
| *RECQL* | BROCA Cancer Risk Panel |
| *RET* | ACMG; BROCA Cancer Risk Panel |
| *RPS20* | BROCA Cancer Risk Panel |
| *SDHA* | NCCN |
| *SDHAF2* | ACMG |
| *SDHB* | ACMG; BROCA Cancer Risk Panel |
| *SDHC* | ACMG; BROCA Cancer Risk Panel |
| *SDHD* | ACMG; BROCA Cancer Risk Panel |
| *SMAD4* | ACMG; BROCA Cancer Risk Panel |
| *SMARCA4* | NCCN; BROCA Cancer Risk Panel |
| *SMARCB1* | NCCN |
| *STK11* | ACMG |
| *SUFU* | NCCN |
| *TMEM127* | ACMG |
| *TP53* | ACMG; BROCA Cancer Risk Panel |
| *TSC1* | ACMG; BROCA Cancer Risk Panel |
| *TSC2* | ACMG; BROCA Cancer Risk Panel |
| *VHL* | ACMG; BROCA Cancer Risk Panel |
| *WT1* | ACMG |
