# Supplementary material for: Germline predisposition and somatic mutational landscape in synchronous mucinous metaplasia and neoplasia of the female genital tract
Source: Clin Transl Med. 2026 Aug 17;16(8):e70768. doi: 10.1002/ctm2.70768 (PMC13478790; doi:10.1002/ctm2.70768)
Supplement: Supplementary file 3 [file CTM2-16-e70768-s004.docx]

| Table S5 Molecular characteristics of pathogenic and likely pathogenic somatic variants of tumor driver genes in SMMN-FGT patients | | | | | | |
| --- | --- | --- | --- | --- | --- | --- |
| Gene | Patient | Consequence | HGVSc | HGVSp | HGVSp_Short | ClinVar |
| *BRAF* | P3.T4 | Missense | c.1799T>A | p.Val600Glu | p.V600E | Likely Pathogenic |
| *CDKN2A* | P3.T4 | Stop Gained | c.79G>T | p.Glu27Ter | p.E27* | Pathogenic |
| *AKT1* | P14.T1 | Missense | c.49G>A | p.Glu17Lys | p.E17K | Likely Pathogenic |
| *TP53* | P14.T1 | Missense | c.743G>A | p.Arg248Gln | p.R248Q | Likely Pathogenic |
| *TP53* | P17.T3 | Stop Gained | c.1024C>T | p.Arg342Ter | p.R342* | Pathogenic |
| *TP53* | P18.T1 | Missense | c.711G>A | p.Met237Ile | p.M237I | Likely Pathogenic |
| *TP53* | P23.T4 | Stop Gained | c.372C>A | p.Cys124Ter | p.C124* | Pathogenic |
