# Supplementary material for: Germline predisposition and somatic mutational landscape in synchronous mucinous metaplasia and neoplasia of the female genital tract
Source: Clin Transl Med. 2026 Aug 17;16(8):e70768. doi: 10.1002/ctm2.70768 (PMC13478790; doi:10.1002/ctm2.70768)
Supplement: Supplementary file 4 [file CTM2-16-e70768-s005.docx]

| Table S3 Pathologic characteristics of the patients | | | | | | | |  |  |
| --- | --- | --- | --- | --- | --- | --- | --- | --- | --- |
| Sample | Age | HPV | Medical history | Cervix | Endometrium | Oviduct | Ovary | Follow-up Time (months) | Clinical Outcome |
| P2 | 37 | （-） |  | gAIS | SGM |  | OMC | 99 | Disease-Free Survival |
| P3 | 41 | （-） |  | GAS | GAS | gAIS | MAC | 15 | Died of Disease |
| P4 | 70 | （-） | Breast cancer resection | GAS | gAIS |  |  | 53 | Disease-Free Survival |
| P6 | 49 | （-） | Short stature（1.52cm), history of diabetes | GAS |  |  | OMC, BMT | 116 | Disease-Free Survival |
| P7 | 54 | （-） | Hypothyroidism | GAS, MDA |  | SGM |  | 41 | Died of Disease |
| P8 | 83 | （-） | Peutz–Jeghers syndrome | LEGH | SGM | SGM |  | 82 | Disease-Free Survival |
| P11 | 39 | （-） | Nasal polypectomy | LEGH | SGM |  |  | 67 | Disease-Free Survival |
| P14 | 52 | （-） |  | GAS | SGM | SGM |  | 45 | Disease-Free Survival |
| P16 | 67 | （+） | Right Achilles tendon repair |  | GAS | GAS |  | 17 | Disease-Free Survival |
| P17 | 67 | （+） | Appendectomy |  | GAS | GAS |  | 15 | Disease-Free Survival |
| P18 | 41 | （-） |  | GAS |  |  | OMC | 30 | Disease-Free Survival |
| P23 | 39 | （-） |  | GAS |  | BMT | BMT, MAC | 127 | Alive |
| P25 | 49 | （-） |  |  |  | BMT | BMT | 14 | Disease-Free Survival |

(-): HPV negative, (+): HPV positive.

GAS: gastric-type mucinous carcinoma; gAIS: gastric-type mucinous carcinoma in situ; MDA: minimal deviation adenocarcinoma; LEGH: lobular endocervical glandular hyperplasia; ALEGH: atypical lobular endocervical glandular hyperplasia; SGM: simple gastric-type mucinous metaplasia; BMT: borderline mucinous tumor; OMC: ovarian mucinous cystadenoma; MAC: mucinous adenocarcinoma.
